# Indoleamine 2,3-dioxygenase 1 and Programmed Cell Death-ligand 1 Co-expression Predicts Poor Pathologic Response and Recurrence in Esophageal Squamous Cell Carcinoma after Neoadjuvant Chemoradiotherapy

**DOI:** 10.3390/cancers11020169

**Published:** 2019-02-01

**Authors:** Sha Zhou, Lei Zhao, Zhaohui Liang, Songran Liu, Yong Li, Shiliang Liu, Hong Yang, Mengzhong Liu, Mian Xi

**Affiliations:** 1 Department of Radiation Oncology, Sun Yat-sen University Cancer Center, State Key Laboratory of Oncology in South China, Collaborative Innovation Centre for Cancer Medicine, Guangdong Esophageal Cancer Institute, Guangzhou 510060, China; zhousha@sysucc.org.cn (S.Z.); zhaolei@sysucc.org.cn (L.Z.); liangzh2@sysucc.org.cn (Z.L.); liushil@sysucc.org.cn (S.L.); liumzh@sysucc.org.cn (M.L.); 2 Department of Pathology, Sun Yat-sen University Cancer Center, State Key Laboratory of Oncology in South China, Collaborative Innovation Centre for Cancer Medicine, Guangdong Esophageal Cancer Institute, Guangzhou 510060, China; liusr@sysucc.org.cn (S.L.); liyong@sysucc.org.cn (Y.L.); 3 Department of Thoracic Oncology, Sun Yat-sen University Cancer Center, State Key Laboratory of Oncology in South China, Collaborative Innovation Centre for Cancer Medicine, Guangdong Esophageal Cancer Institute, Guangzhou 510060, China; yanghong@sysucc.org.cn

**Keywords:** esophageal squamous cell carcinoma, neoadjuvant chemoradiotherapy, IDO1, PD-L1, pathologic response

## Abstract

This study aimed to investigate the impact of indoleamine 2,3-dioxygenase 1 (IDO1) expression, programmed cell death-ligand 1 (PD-L1) expression, CD8+ tumor-infiltrating lymphocyte (TIL) status, and their combination on pathologic complete response (pCR) and recurrence in esophageal squamous cell carcinoma (ESCC) treated with neoadjuvant chemoradiotherapy (CRT). Indoleamine 2,3-dioxygenase 1, PD-L1, and CD8+ TIL statuses were evaluated by immunohistochemical analysis on pre-CRT biopsies of 158 patients. Sixty-eight patients (43.0%) achieved pCR after neoadjuvant CRT and 48 patients (30.4%) developed recurrences after surgery. IDO1 and PD-L1 proteins were co-expressed in 28 patients (17.7%). Indoleamine 2,3-dioxygenase 1 positive patients showed a significantly lower pCR rate than IDO1 negative patients (28.6% vs. 51.0%, *P* = 0.007). Similarly, PD-L1 high expression was significantly negatively correlated with pCR rate (27.3% vs. 51.5%, *P* = 0.004). On multivariate analysis, IDO1 expression was an independent prognostic factor for developing recurrences. Stratification analysis revealed that patients with co-expression of IDO1 and PD-L1 were significantly associated with a lower pCR rate and worse recurrence-free survival than those with one or none positive protein. In conclusion, IDO1 and PD-L1 co-expression could predict poor pathologic response and high risk of recurrence in ESCC after neoadjuvant CRT, indicating a subset of patients who may benefit from CRT combined with immunotherapy.

## 1. Introduction

Esophageal cancer (EC) is a significant heath burden worldwide, with an estimated 572,034 new cases and 508,585 deaths each year [1]. For locally advanced EC, neoadjuvant chemoradiotherapy (CRT) followed by esophagectomy has become the standard treatment option for both adenocarcinoma and squamous cell carcinoma (SCC) subtypes [2,3,4]. When employed, neoadjuvant CRT results in pathologic complete response (pCR) rates of 20% to 49%, which have been linked to significantly improved long-term survival benefit [3,4,5,6]. As no major advances in the improvement of pCR rates after neoadjuvant CRT have been made in recent years, there is critical clinical need to search for innovative strategies to improve efficacy for EC patients.

Immunotherapy has rapidly emerged as a novel treatment option and has changed the landscape of therapy for many types of malignancy, including EC [7]. Programmed cell death-1 (PD-1)/programmed cell death-ligand 1 (PD-L1) pathway is one of the most important signaling pathways that mediate tumor immune escape [8]. Although a series of clinical trials have shown promising antitumor efficacy of PD-1/PD-L1 blockade, only approximately 12% to 30% of EC patients can achieve favorable response and durable efficacy, suggesting that combining PD-1/PD-L1 inhibition with chemotherapy, radiotherapy, or other immune checkpoint inhibitors should be explored [9,10,11,12].

As a rate-limiting enzyme in the metabolism of essential amino acid tryptophan in the peripheral tissue, indoleamine 2,3-dioxygenase 1 (IDO1) is another promising target for cancer therapy [13]. With immunosuppressive properties, IDO1 activation leads to suppression of cytotoxic T cell function by promoting cell cycle arrest and apoptosis. Furthermore, IDO1 inhibits the function of natural killer cells and promotes the activation of myeloid-derived suppressor cells and dendritic cells. Additionally, IDO1 can enhance the activity of regulatory T cells which further downregulate the activity of effector T cells and natural killer cells [13,14]. A recent study showed that IDO1 activity can be involved in resistance to anti-PD-1 treatment in lung cancer [15]. Accordingly, phase 1/2 trials have indicated that the combination of IDO1 and PD-1 inhibitors may improve patient responses to PD-1 inhibitors alone [16,17].

Previous studies have revealed that immune checkpoints such as IDO1 and PD-L1 are overexpressed in EC, which are associated with poor clinical outcomes in patients receiving surgery alone [18,19,20]. However, whether the immunologic phenotype is relevant for pathologic response to neoadjuvant CRT remains unknown. Moreover, the association between IDO1 and PD-L1 is also unclear. Therefore, this study aimed to investigate the impact of IDO1 expression, PD-L1 expression, CD8+ tumor-infiltrating lymphocyte (TIL) status, and their combination on pathologic response and recurrence in EC patients who underwent neoadjuvant CRT. Owing to the rarity of esophageal adenocarcinoma in China, this study focused only on esophageal squamous cell carcinoma (ESCC). 

## 2. Results

### 2.1. Patient Characteristics

Patient and treatment characteristics of 158 ESCC patients who met inclusion criteria are summarized in Table 1. Median age of this cohort was 56 years (range, 42–73 years) and the majority of patients had clinical stage III disease (78.5%). The median radiation dose was 40.0 Gy (range, 36.0–50.4 Gy). Patients underwent esophagectomy within a median interval of 46 days (range, 29–92 days) after neoadjuvant CRT. After histopathological examination, 68 patients (43.0%) achieved a pCR.

### 2.2. Quantitative Reverse Transcription Polymerase Chain Reaction

**Figure 1:**
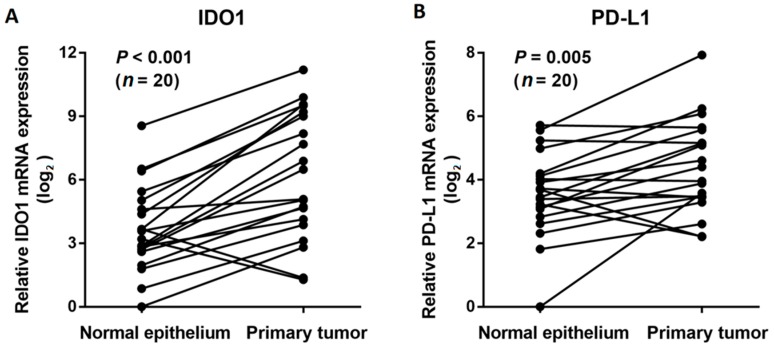
Comparison of indoleamine 2,3-dioxygenase 1 (IDO1) (**A**) and programmed cell death-ligand 1 (PD-L1) (**B**) mRNA expression levels in esophageal squamous cell carcinoma tissues and matched normal esophageal mucosa by qRT-PCR.

The mRNA expression levels of IDO1 and PD-L1 were examined by quantitative reverse transcription polymerase chain reaction (PCR) in 20 frozen ESCC tissues and matched normal epithelium. As expected, tumor tissues expressed significantly higher levels of IDO1 than normal mucosa (*P* < 0.001, Figure 1A). Similar to IDO1, the PD-L1 mRNA expression levels were also notably higher in tumor tissues than in normal epithelium (*P* = 0.005, Figure 1B). 

### 2.3. Correlation of Indoleamine 2,3-Dioxygenase 1 and Programmed Cell Death-Ligand 1 Expression with Clinicopathologic Characteristics

**Figure 2:**
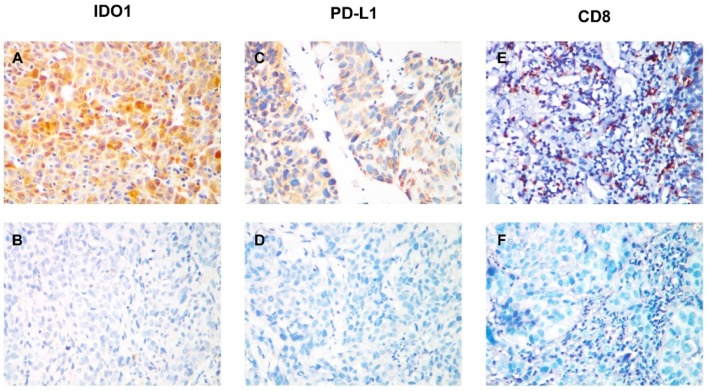
IDO1 and PD-L1 expression and CD8+ tumor-infiltrating lymphocyte (TIL) status in esophageal squamous cell carcinoma. (**A**) Positive immunohistochemical staining pattern for IDO1; (**B**) Negative immunohistochemical staining pattern for IDO1; (**C**) Positive immunohistochemical staining pattern for PD-L1; (**D**) Negative immunohistochemical staining pattern for PD-L1; (**E**) Pattern for high CD8+ TIL density; (**F**) Pattern for low CD8+ TIL density.

According to IHC staining, IDO1 and PD-L1 proteins were positively expressed in 56 (35.4%) and 55 (34.8%) patients, respectively. The median CD8 density was 18 (range, 0–106) in the whole cohort, and 80 (50.6%) patients were classified as CD8 high density group. Representative IDO1, PD-L1, and CD8 staining patterns are shown in Figure 2. As listed in Table 2. Indoleamine 2,3-dioxygenase 1 positivity was significantly associated with alcohol history, longer primary tumor, and advanced tumor stage, whereas PD-L1 positivity was significantly correlated with smoking history. Moreover, a significant correlation was observed between IDO1 and PD-L1 expression (*P* = 0.003).

### 2.4. Factors Associated with Pathologic Complete Response 

**Figure 3:**
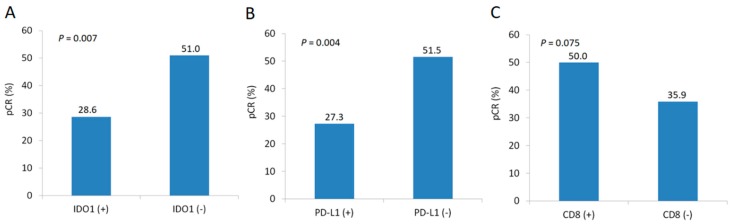
Comparison of pathologic complete response rates by IDO1 expression status (**A**), PD-L1 expression status (**B**), and CD8 density (**C**).

We identified patient and treatment characteristics associated with pCR (Table 3). IDO1 positive patients showed a significantly lower pCR rate than IDO1 negative patients (28.6% vs. 51.0%, *P* = 0.007; Figure 3A). Likely, PD-L1 high expression was significantly negatively correlated with pCR rate (27.3% vs. 51.5%, *P* = 0.004; Figure 3B). A marginally significant correlation between CD8 density and pCR was also observed (50.0% vs. 35.9%, *P* = 0.075; Figure 3C). On multivariate analysis, IDO1 and PD-L1 expression remained significantly associated with pCR (IDO1: odds ratio 2.194, *P* = 0.032; PD-L1: odds ratio 2.425, *P* = 0.017).

### 2.5. Survival Analysis

At the time of analysis, the median follow-up time for survivors was 40.2 months (range, 1.1–176.1 months). A total of 45 patients (28.5%) had died and 48 (30.4%) developed recurrences during follow-up. Sixteen patients (10.1%) developed locoregional failure only, 25 (15.8%) had distant failure only, and 7 (4.4%) experienced concomitant locoregional and distant recurrences. The 3-year overall survival (OS) rate and 3-year recurrence-free survival (RFS) rate for the whole cohort were 72.1% and 72.3%, respectively.

**Figure 4:**
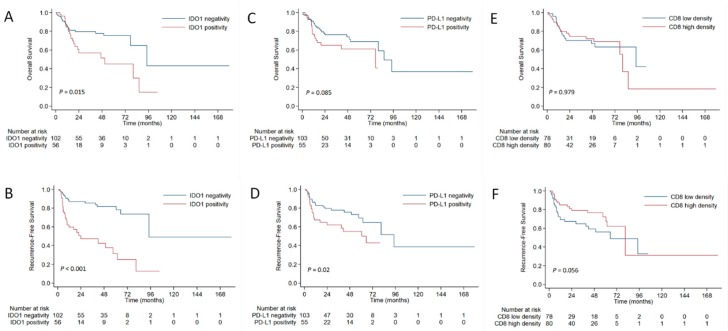
Comparison of overall survival (**A**) and recurrence-free survival (**B**) between patients with positive or negative IDO1 expression. Comparison of overall survival (**C**) and recurrence-free survival (**D**) between patients with positive or negative PD-L1 expression. Comparison of overall survival (**E**) and recurrence-free survival (**F**) between patients with high or low CD8 density.

Patients with IDO1 positivity demonstrated a significantly higher recurrence rate than those with IDO1 negativity (53.6% vs. 17.6%, *P* < 0.001), and PD-L1 positivity was also correlated with recurrence risk (41.8% vs. 24.3%, *P* = 0.022). Comparing with IDO1 negativity, IDO1 positivity was significantly associated with worse OS and RFS (Figure 4A,B). The PD-L1 expression and CD8 density were significant prognostic factors for RFS but not for OS (Figure 4C–F). Multivariate analysis revealed that age, chemotherapy regimen, and IDO1 expression were independent prognostic factors for developing recurrences (Table 4).

### 2.6. Stratification Analysis

**Figure 5:**
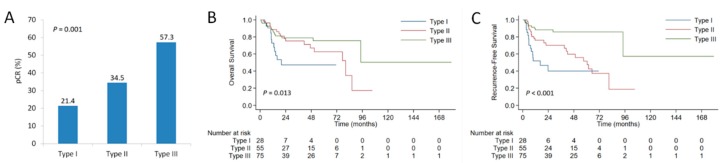
Comparison of pathologic complete response rates according to the co-expression status of IDO1 and PD-L1 (**A**). Kaplan-Meier curves for overall survival (**B**) and recurrence-free survival (**C**) in patients with esophageal squamous cell carcinoma according to the co-expression status of IDO1 and PD-L1. Type I: IDO1 (+)/PD-L1 (+); Type II: IDO1 (−)/PD-L1 (+) or IDO1 (+)/PD-L1 (−); Type III: IDO1 (−)/PD-L1 (−).

Of the 158 patients, 28 (17.7%) were IDO (+)/PD-L1 (+), 28 (17.7%) were IDO (+)/PD-L1 (−), 27 (17.1%) were IDO (−)/PD-L1 (+), and 75 (47.5%) were IDO (−)/PD-L1 (−). Considering the positive correlation between IDO1 and PD-L1 expression, we divided patients into 3 immune subtypes: IDO (+)/PD-L1 (+), IDO (+)/PD-L1 (−) or IDO (−)/PD-L1 (+), and IDO (−)/PD-L1 (−), with corresponding pCR rates of 21.4%, 34.5%, and 57.3%, respectively (*P* = 0.001; Figure 5A). In terms of survival endpoints, the IDO (+)/PD-L1 (+) group demonstrated significantly worse OS and RFS than the other two groups (Figure 5B,C). The 3-year RFS rates were 40.0% for IDO (+)/PD-L1 (+) group, 70.2% for IDO (+)/PD-L1 (−) or IDO (−)/PD-L1 (+) group, and 85.8% for IDO (−)/PD-L1 (−) group, respectively (*P* < 0.001).

## 3. Discussion

Although targeting immune checkpoints has shown therapeutic activity in EC, the correlation of tumoral immune status with pathologic response to neoadjuvant CRT remains unclear. In this study, we found that the co-expression of IDO1 and PD-L1 could be not only a predictor for poor pathologic response but also a prognostic factor for high risk of recurrence in ESCC after neoadjuvant CRT. These findings are very important for defining risk-adapted therapeutic strategies and also helpful to select appropriate immunotherapy regimens combining with CRT and surgery.

Previous investigations have consistently demonstrated that baseline PD-L1 positivity and higher density of TILs are related to better chemotherapy response in breast cancer [21,22]. Teng et al. reported that patients with high density of CD8+ and CD4+ TILs were more likely to achieve good pathologic response to neoadjuvant CRT in rectal cancer [23]. However, a retrospective study with a small sample size of 31 esophageal adenocarcinoma revealed no association between pretreatment PD-L1 status and pathologic response to neoadjuvant therapy [24]. Based on a relatively large cohort of patients, our results indicated that both IDO1 and PD-L1 expression were negatively correlated with pCR after neoadjuvant CRT in ESCC. Nevertheless, the correlation between CD8 density and pCR was not significant on multivariate analysis. These observations suggest that the clinical significance of immune checkpoints may vary in different types of cancer.

The prognostic role of PD-L1 expression and TIL status for survival in patients with SCC are still controversial in clinical practice [25,26]. For EC patients treated with surgery alone, Yagi et al. demonstrated that PD-L1 positivity and CD8+ TIL status were associated with significantly worse OS [18]. On the contrary, Hatogai et al. reported that PD-L1 expression in both tumor cells and TILs were notably associated with favorable OS in 196 ESCC patients who received curative resection alone [27]. We also investigated whether IDO1, PD-L1, and CD8 status were prognostic markers for survival in this study. Neither PD-L1 expression nor CD8 density was a significant prognostic factor for OS in our results, indicating the effect of immune microenvironment on survival may vary with treatment strategies.

Given only a minority of patients can benefit from immunotherapy, searching for predictive biomarkers to guide treatment decisions has prompted interest in clinical practice. Based on different expression status of IDO1 and PD-L1, patients were classified into 3 immune subtypes with different prognostic features in the current study, which had important implications to develop individualized treatment strategies. Of them, IDO1 and PD-L1 proteins were co-expressed in 17.7% of patients. Kozuma et al. reported that both IDO1 and PD-L1 expression were upregulated by interferon (IFN)-γ and tumor growth factor (TGF)-β in lung adenocarcinoma, leading to an evasion of host immune responses [28]. Accordingly, IDO1 was infrequently expressed in isolation but was more frequently co-expressed in cases with PD-L1 positivity, which has been confirmed in thyroid carcinoma as well [29]. Consistent with previous reports, IDO1 expression was significantly correlated with PD-L1 expression in our results. Since the immune response of PD-1/PD-L1 inhibition might be limited by the upregulation of IDO1 [15], the dual blockade of PD-L1 and IDO1 may be needed to improve efficacy for patients with co-expression of IDO1 and PD-L1. Patients with IDO (−)/PD-L1 (+) or IDO (+)/PD-L1 (−) are most likely to benefit from a single anti-PD-1/PD-L1 blockade or anti-IDO1 blockade. For patients with IDO (−)/PD-L1 (−), immunotherapy might be of less value. Given the high pCR rate (57.3%) and the remarkably low risk of recurrence in this type, stratification of patients who would benefit from radical esophagectomy versus active surveillance should be studied.

Growing evidences have documented the synergism between immunotherapy and radiotherapy [30,31]. Through a cytotoxic T cell-dependent mechanism, the combination of anti-PD-L1 therapy and radiotherapy significantly inhibited tumor growth compared with radiotherapy alone or anti-PD-L1 monotherapy in mice [30,31,32,33]. Similarly, Li et al. reported that IDO inhibitor synergized with CRT to prolong survival against murine glioblastoma [34]. Moreover, a preclinical study found that IDO1 had an independent influence on tumor cell’s resistance to radiation in the absence of immune cells, including effects on DNA repair and depletion of cells in G2/M of the cell cycle [35]. Preclinical studies also demonstrated that radiotherapy could upregulate the expression of PD-L1 and IDO1 in tumor cells, which has been confirmed in human EC tissues [24,36]. However, it should be noted that the upregulation of PD-L1 and IDO1 is dose-dependent and transiently elevated post radiation in animal model [24,32]. Therefore, concurrent fractionated radiotherapy with PD-1/PD-L1 and IDO1 inhibition but not sequential administration may bring more therapeutic benefit to patients. Ladomersky et al. recently demonstrated a durable survival benefit from this novel three-agent combination in mice with advanced glioblastoma, but not for any single- or dual-agent combination [37]. Our study indicated that pathologic response of ESCC to CRT is at least partly mediated by immune microenvironment, particularly IDO1 and PD-L1 expression. Theoretically, IDO1 and PD-1/PD-L1 inhibitors combined with radiotherapy might be a potentially promising regimen to enhance radiosensitivity of ESCC.

This study has several limitations. First, the results may be influenced by the selection bias due to its retrospective nature from a single institution; thus, validation should be performed by prospective studies as well as external cohorts. Second, the expression of immune checkpoints was evaluated from small samples obtained by endoscopic biopsy, thus we cannot differentiate the central tumor from the invasion front. Third, we performed IDO1 IHC assay using a single monoclonal antibody. Since no guidelines for antibody use or quantifying IDO1 expression in ESCC have been defined in the literature, the reproducibility of our results should be validated in future.

## 4. Patients and Methods

### 4.1. Patients

All ESCC patients who received neoadjuvant CRT followed by surgery from the prospectively maintained database at our institution between January 2003 and December 2016 were retrospectively analyzed. The eligibility criteria included: pre-CRT biopsy obtained, pathologic confirmation of stage II-III ESCC according to the 7th TNM staging system of the American Joint Committee on Cancer, and completion of neoadjuvant CRT followed by esophagectomy with curative intent. This study was approved by the Ethics Committee of Sun Yat-sen University Cancer Center (No. B2019-010-01) and informed consent was waived due to its retrospective nature.

All patients received external-beam radiation using three-dimensional conformal radiotherapy (3DCRT) or intensity-modulated radiotherapy (IMRT). The typical prescribed dose was 40.0–45.0 Gy with a daily fraction of 1.8–2.0 Gy. All patients received concurrent platinum-based chemotherapy during radiation and esophagectomy was performed approximately 6 to 8 weeks after the completion of CRT. The pCR was defined as no viable cancer cells in all layers of the esophagus and in the lymph nodes resected. After surgery, patients were followed every 3 months during the first year, then every 6 months for the next 2 years, and thereafter annually. The first recurrence pattern was recorded to classify locoregional or distant recurrence, which was established on histologic, cytologic, or explicit radiologic proof.

### 4.2. Quantitative Reverse Transcription Polymerase Chain Reaction

Total RNA from primary ESCC tissues and the adjacent normal tissues obtained from the same patient was extracted using TRIzol reagent (Invitrogen, Carlsbad, CA, USA) according to the manufacturer’s protocol. Then, reverse transcription of total RNA was performed to synthesize cDNA, which was amplified and quantified by SYBR-Green in CFX96 Real Time System C1000 Cycler (Bio-Rad Laboratories, Singapore). Relative quantification of gene expression levels were analyzed by the 2^−ΔΔC^_T_ method and GAPDH was used for normalization. Each experiment was repeated at least three times in triplicate. The primer sequences used in real-time PCR were as follows: IDO1 (Forward, 5′-GCCTGATCTCATAGAGTCTGGC-3′ and Reverse, 5′-TGCATCCCAGAACTAGACGT GC-3′), PD-L1 (Forward, 5′-TGCCGACTACAAGCGAATTACTG-3′ and Reverse, 5′-CTGCTTGTCC AGATGACTTCGG-3′), and GAPDH (Forward, 5′-GTCTCCTCTGACTTCAACAGCG-3′ and Reverse, 5′-ACCACCCTGTTGCTGTAGCCAA-3′).

### 4.3. Immunohistochemistry

Formalin-fixed and paraffin-embedded tumor tissues were cut into 4-µm-thick sections for immunohistochemical (IHC) analysis. After deparaffinization, antigen was retrieved by citric acid buffer (pH 6.0) using a steamer autoclave, then incubated with anti-IDO1 antibody (HPA023072, Sigma, Shanghai, China) at 1:1000 dilution, anti-PD-L1 antibody (ab213524, Abcam, Cambridge, MA, USA) at 1:250 dilution, and anti-CD8 antibody (ab108343, Abcam) at 1:500 dilution overnight at 4 °C. Subsequently, the sections were incubated with a secondary antibody, stained with diaminobenzidine, and then counterstained with hematoxylin, dehydrated, and coverslipped.

All IHC analyses were evaluated by two experienced pathologists (Y.L. and S.L.) who were unaware of patient clinical information. Expression of IDO1 in tumor tissues were considered positive if tumor cytoplasmic and membrane staining >50% regardless of intensity. For PD-L1 staining, >1% tumor membranous staining was considered positive and PD-L1 expression on tumor-infiltrating immune cells was not scored. The quantitative density of CD8+ TILs was evaluated within 5 stromal areas of the tumor under high-power magnification of 400× for each patient. The average number of CD8+ TILs per high power field was counted and the median number of CD8+ TILs was determined as the cut-off point for CD8 density.

### 4.4. Statistical Analysis

Associations between IDO1 and PD-L1 expression, CD8+ TIL status, and patient clinicopathologic features were examined using the chi-square or Fisher’s exact tests. Follow-up and survival times were defined from the date of surgery until event or censor. Kaplan-Meier method was used to estimate OS and RFS. Log-rank test was used to examine intergroup differences, and Cox proportional hazards regression model was used to analyze prognostic factors for disease recurrence (backward stepwise). Univariate and multivariate logistic regression models were performed to analyze possible predictors of pCR. Variables with *P* ≤ 0.1 in the univariate analysis were subjected to the multivariate analysis. Statistical analyses were performed using SPSS 22.0 software (SPSS Inc., Chicago, IL, USA). *P* < 0.05 was considered statistically significant.

## 5. Conclusions

Indoleamine 2,3-dioxygenase 1 and PD-L1 co-expression could predict poor pathologic response and high risk of recurrence in ESCC after neoadjuvant CRT, indicating a subset of patients who may benefit from CRT combined with immunotherapy. Future prospective studies are warranted to confirm its role as a predictive biomarker for pCR as well as an indicator for the selection of immunotherapy regimens.

## Figures and Tables

**Table 1:** Patient characteristics.

Characteristic	Total (*n* = 158), %
Age (years)	
<56	72 (45.6)
≥56	86 (54.4)
Sex	
Male	131 (82.9)
Female	27 (17.1)
Smoking history	
Yes	104 (65.8)
No	54 (34.2)
Alcohol history	
Yes	57 (36.1)
No	101 (63.9)
Performance status	
0	90 (57.0)
1–2	68 (43.0)
Weight loss	
<10%	140 (88.6)
≥10%	18 (11.4)
Histologic grade	
Gx/1/2	118 (74.7)
G3	40 (25.3)
Tumor location	
Upper/middle	120 (75.9)
Distal	38 (24.1)
Primary tumor length	
≤5 cm	72 (45.6)
>5 cm	86 (54.4)
Clinical T stage	
T1-2	30 (19.0)
T3-4	128 (81.0)
Clinical N stage	
N0	11 (7.0)
N1-3	147 (93.0)
Clinical TNM stage	
II	34 (215)
III	124 (78.5)
Chemotherapy regimen	
Cisplatin/vinorelbine	92 (58.2)
Cisplatin/fluorouracil	16 (10.1)
Cisplatin/taxane	50 (31.6)
Radiation dose (Gy)	
Median (range)	40.0 (36.0–50.4)
Radiotherapy modality	
3DCRT	105 (66.5)
IMRT	53 (33.5)

Abbreviations: 3DCRT, three-dimensional conformal radiation therapy; IMRT, intensity-modulated radiation therapy.

**Table 2:** Relationship between IDO1 and PD-L1 expression and patient clinicopathological features.

Characteristic	IDO1 Expression		*P*-Value	PD-L1 Expression		*P*-Value
	Negative	Positive		Negative	Positive	
Age (years)			0.135			0.098
<56	42 (41.2%)	30 (53.6%)		42 (40.8%)	30 (54.5%)	
≥56	60 (58.8%)	26 (46.4%)		61 (59.2%)	25 (45.5%)	
Sex			0.115			0.132
Male	81 (79.4%)	50 (89.3%)		82 (79.6%)	49 (89.1%)	
Female	21 (20.6%)	6 (10.7%)		21 (20.4%)	6 (10.9%)	
Smoking history			0.690			0.041
Yes	66 (64.7%)	38 (67.9%)		62 (60.2%)	42 (76.4%)	
No	36 (35.3%)	18 (32.1%)		41 (39.8%)	13 (23.6%)	
Alcohol history			0.045			0.687
Yes	31 (30.4%)	26 (46.4%)		36 (35.0%)	21 (38.2%)	
No	71 (69.6%)	30 (53.6%)		67 (65.0%)	34 (61.8%)	
Performance status			0.480			0.573
0	56 (54.9%)	34 (60.7%)		57 (55.3%)	33 (60.0)	
1–2	46 (45.1%)	22 (39.3%)		46 (44.7%)	22 (40.0%)	
Weight loss			0.213			0.700
<10%	88 (86.3%)	52 (92.9%)		92 (89.3%)	48 (87.3%)	
≥10%	14 (13.7%)	4 (7.1%)		11 (10.7%)	7 (12.7%)	
Histologic grade			0.405			0.679
Gx/1/2	74 (72.5%)	44 (78.6%)		78 (75.7%)	40 (72.7%)	
G3	28 (27.5%)	12 (21.4%)		25 (24.3%)	15 (27.3%)	
Tumor location			0.078			0.763
Upper/middle	82 (80.4%)	38 (67.9%)		79 (76.7%)	41 (74.5%)	
Distal	20 (19.6%)	18 (32.1%)		24 (23.3%)	14 (25.5%)	
Primary tumor length			0.012			0.090
≤5 cm	54 (52.9%)	18 (32.1%)		52 (50.5%)	20 (36.4%)	
>5 cm	48 (47.1%)	38 (67.9%)		51 (49.5%)	35 (63.6%)	
Clinical T stage			0.123			0.850
T1-2	23 (22.5%)	7 (12.5%)		20 (19.4%)	10 (18.2%)	
T3-4	79 (77.5%)	49 (87.5%)		83 (80.6%)	45 (81.8%)	
Clinical N stage			0.330			0.332
N0	9 (8.8%)	2 (3.6%)		9 (8.7%)	2 (3.6%)	
N1-3	93 (91.2%)	54 (96.4%)		94 (91.3%)	53 (96.4%)	
Clinical TNM stage			0.041			0.734
II	27 (26.5%)	7 (12.5%)		23 (22.3%)	11 (20.0)	
III	75 (73.5%)	49 (87.5%)		80 (77.7%)	44 (80.0)	
CD8+ TIL density			0.264			0.777
Low	47 (46.1%)	31 (55.4%)		50 (48.5%)	28 (50.9%)	
High	55 (53.9%)	25 (44.6%)		53 (51.5%)	27 (49.1%)	

Abbreviations: IDO1, indoleamine 2,3-dioxygenase 1; PD-L1, programmed cell death-ligand 1; TIL, tumor-infiltrating lymphocyte.

**Table 3:** Univariate and multivariate analyses for variables associated with pathologic complete response.

Variable	Univariate		Multivariate	
	Odds Ratio (95% CI)	*P*-Value	Odds Ratio (95% CI)	*P*-Value
Age (<56 vs. ≥56)	1.001 (0.532–1.884)	0.997		
Sex (female vs. male)	1.840 (0.798–4.242)	0.153		
Smoking history (yes vs. no)	0.580 (0.299–1.127)	0.108		
Alcohol history (yes vs. no)	0.597 (0.306–1.166)	0.131		
Performance status (0 vs. 1–2)	0.750 (0.397–1.417)	0.375		
Weight loss (<10% vs. ≥10%)	0.728 (0.273–1.947)	0.527		
Histologic grade (Gx/1/2 vs. G3)	1.182 (0.570–2.451)	0.654		
Tumor location (upper/middle vs. distal)	1.051 (0.503–2.200)	0.894		
Primary tumor length (≤5 vs. >5 cm)	1.233 (0.655–2.321)	0.516		
Clinical TNM stage (II vs. III)	1.231 (0.574–2.637)	0.593		
Chemotherapy regimen^a^ (1 vs. 2/3)	1.790 (0.934–3.431)	0.079		
Radiation dose (≤40 vs. >40 Gy)	0.849 (0.429–1.681)	0.639		
Radiotherapy modality (3DCRT vs. IMRT)	1.235 (0.631–2.417)	0.538		
IDO1 (negative vs. positive)	2.600 (1.294–5.224)	0.007	2.194 (1.068–4.507)	0.032
PD-L1 (negative vs. positive)	2.827 (1.392–5.739)	0.004	2.425 (1.172–5.021)	0.017
CD8+ TIL density (low vs. high)	0.560 (0.296–1.059)	0.075		

Abbreviations: CI, confidence interval; 3DCRT, three-dimensional conformal radiation therapy; IMRT, intensity-modulated radiation therapy; IDO1, indoleamine 2,3-dioxygenase 1; PD-L1, programmed cell death-ligand 1; TIL, tumor-infiltrating lymphocyte. ^a^ Chemotherapy regimen: 1, cisplatin/vinorelbine; 2, cisplatin/fluorouracil; 3, cisplatin/taxane.

**Table 4:** Univariate and multivariate analyses for recurrence-free survival.

Variable	Univariate		Multivariate	
	Hazard Ratio (95% CI)	*P*-Value	Hazard Ratio (95% CI)	*P*-Value
Age (<56 vs. ≥56)	3.585 (1.894–6.788)	<0.001	3.332 (1.744–6.369)	<0.001
Sex (female vs. male)	0.280 (0.087–0.902)	0.033		
Smoking history (yes vs. no)	0.812 (0.435–1.516)	0.514		
Alcohol history (yes vs. no)	0.689 (0.389–1.221)	0.202		
Performance status (0 vs. 1–2)	1.061 (0.600–1.876)	0.838		
Weight loss (<10% vs. ≥10%)	0.993 (0.391–2.519)	0.988		
Histologic grade (Gx/1/2 vs. G3)	1.241 (0.615–2.501)	0.547		
Tumor location (upper/middle vs. distal)	1.226 (0.611–2.460)	0.567		
Primary tumor length (≤5 vs. >5 cm)	0.606 (0.336–1.090)	0.095		
Clinical TNM stage (II vs. III)	0.588 (0.263–1.314)	0.196		
Chemotherapy regimen ^a^ (1 vs. 2/3)	0.464 (0.258–0.837)	0.011	0.420 (0.228–0.775)	0.005
Radiation dose (≤40 vs. >40 Gy)	1.121 (0.524–2.396)	0.769		
Radiotherapy modality (3DCRT vs. IMRT)	1.255 (0.586–2.687)	0.559		
IDO1 (negative vs. positive)	0.236 (0.131–0.427)	<0.001	0.282 (0.153–0.519)	<0.001
PD-L1 (negative vs. positive)	0.509 (0.285–0.907)	0.022		
CD8+ TIL density (low vs. high)	1.748 (0.978–3.126)	0.060		
Pathologic response (pCR vs. non-pCR)	0.401 (0.211–0.764)	0.005		

Abbreviations: CI, confidence interval; 3DCRT, three-dimensional conformal radiation therapy; IMRT, intensity-modulated radiation therapy; IDO1, indoleamine 2,3-dioxygenase 1; PD-L1, programmed cell death-ligand 1; TIL, tumor-infiltrating lymphocyte; pCR, pathologic complete response. ^a^ Chemotherapy regimen: 1, cisplatin/vinorelbine; 2, cisplatin/fluorouracil; 3, cisplatin/taxane.
